# The Effect of Steep Trendelenburg Positioning on Retinal Structure and Function during Robotic-Assisted Laparoscopic Procedures

**DOI:** 10.1155/2018/1027397

**Published:** 2018-06-13

**Authors:** Kazuyuki Hirooka, Kaori Ukegawa, Eri Nitta, Nobufumi Ueda, Yushi Hayashida, Hiromi Hirama, Rikiya Taoka, Yuma Sakura, Mari Yamasaki, Hiroyuki Tsunemori, Mikio Sugimoto, Yoshiyuki Kakehi

**Affiliations:** ^1^Department of Ophthalmology, Kagawa University Faculty of Medicine, 1750-1 Ikenobe, Miki, Kagawa 761-0793, Japan; ^2^Department of Urology, Kagawa University Faculty of Medicine, 1750-1 Ikenobe, Miki, Kagawa 761-0793, Japan

## Abstract

**Purpose:**

Robotic-assisted laparoscopic radical prostatectomy (RALP) has become a standard treatment choice for localized prostate cancer. RALP requires a steep Trendelenburg position, which leads to a significant increase in intraocular pressure (IOP). This study evaluated the effect on the retinal structure and function in patients undergoing RALP.

**Methods:**

Standard automated perimetry (SAP) and optical coherence tomography (OCT) were performed in 20 males scheduled for RALP at 1 month and 1 day before the operation and at 1 and 3 months after the operation. IOP measurements were made in the supine position at 5 min after intubation under general anesthesia (T1), at 6 discrete time points (5, 30, 60, 120, 180, and 240 min; T2-7), and at 5 min after returning to a horizontal supine position (T8). Serial retinal nerve fiber layer (RNFL) thicknesses and visual field progression were assessed using the guided progression analysis software program. RNFL thickness progression and visual field progression were evaluated by event analysis.

**Results:**

Average IOP (mmHg) for each time point was as follows: T1 = 12.3 ± 2.6, T2 = 20.4 ± 4.2, T3 = 23.3 ± 3.8, T4 = 24.0 ± 3.2, T5 = 24.3 ± 3.4, T6 = 27.1 ± 7.2, T7 = 29.8 ± 8.7, and T8 = 20.1 ± 4.4. During RALP, IOP significantly increased. There was no progression of the visual field and RNFL thickness after surgery or any other ocular complications found.

**Conclusions:**

Although IOP significantly increased during RALP, there were no significant changes in the retinal structure and function between the pre- and postoperation observations.

## 1. Introduction

Prostate cancer is one of the most common cancers in men. Although there are several treatment options, radical prostatectomy is a standard treatment for clinically localized prostate cancer. After introduction of robotic-assisted laparoscopic radical prostatectomy (RALP), it has spread rapidly in the world. As compared to the radical prostatectomy, there are several benefits associated with RALP, including reduced blood loss, fewer perioperative complications, improved functional outcomes, and a faster return to work [1–3].

RALP requires the use of a steep Trendelenburg position in which patients are placed in a supine position with their feet positioned above their head at an angle of inclination of 25 to 30 degrees. However, an increased intraocular pressure (IOP) has been reported to occur during surgeries when using the steep Trendelenburg position [4–8]. After refractive surgeries, it has also been reported that increases in the IOP during the operation can potentially lead to complications such as glaucoma and ischemic optic neuropathy [9, 10]. Hoshikawa et al. previously reported that despite finding a significantly increased IOP during the RALP procedure, they did not observe any significant changes in the retinal nerve fiber layer (RNFL) thickness or visual acuity [5]. In contrast, Taketani et al. recently reported OCT-detected visual field defects at 1 week after surgery, even though they found no abnormal findings in the fundus, RNFL thickness, or optic disc morphology, with the visual field returning to normal within 3 months after the surgeries in all cases [7]. It should be noted, however, that since perimetry is a psychophysical test, this technique will be limited by the “noise” of the variability, which is dependent upon the nature of the changes in the visual system, the testing situation, and the patient's condition [11]. Changes outside the limits of the short-term variability can be identified by guided progression analysis (GPA), which is a statistical method that uses the analysis of variance [12].

The aim of our current study was to investigate the influence of RALP on the retinal function and structure through the use of GPA software in patients without preexisting ocular disease.

## 2. Materials and Methods

### 2.1. Patients

This single-center, prospective, nonrandomized study was conducted in accordance with the principles outlined in the Declaration of Helsinki. The Ethics Committee of Kagawa University Faculty of Medicine approved the study protocol. After explanation of the study protocol, each subject provided written informed consent. Between March and September of 2016 at Kagawa University Hospital, we enrolled a total of 24 consecutive male patients who underwent the RALP procedure. Enrolled patients were evaluated at our Ophthalmology Department at 1 month and 1 day prior to the operation and at 1 and 3 months after the operation. Since local visual field defects were recovering to normal within 3 months after the operation in the previous study [7], we evaluated glaucomatous progression until 3 months after the operation. Each of the subjects underwent ophthalmic evaluations at both of the visits. The examinations performed included dilated fundus examination with stereoscopic biomicroscopy of the optic nerve head using slit-lamp and indirect ophthalmoscopy, IOP testing, and visual acuity testing with refraction. Subjects also underwent gonioscopic examinations, which were performed and evaluated by the Shaffer classification. Subjects with previous abnormal visual field test results, ocular hypertension (IOP >21 mmHg), or the history of any kind of neurologic disease, retinal laser procedure, retinal pathology, or retinal surgery were excluded from the study. All subjects underwent visual field and optical coherence tomography (OCT) testing.

### 2.2. Cirrus HD-OCT RNFL Thickness and Optic Disc Morphology

Cirrus HD-OCT (Carl Zeiss Meditec, Dublin, CA), which is based on the use of spectral domain technology, was used to obtain all measurements by using an optic disc cube that was generated from a 3-dimensional data set. Each data set was composed of 200 A-scans from each of 200 B-scans that were obtained over a 6 mm^2^ area that was centered on the optic disc. The methods for measuring and analyzing the RNFL thickness have been previously described elsewhere in detail [4]. The cube is used to create an RNFL thickness map, with the software then determining the center of the disc. Using this data set, the software subsequently extracts a circumpapillary circle (1.73 mm radius). To be included in the analysis of the current study, the images were required to have signal strengths of at least 6. The RNFL thickness deviation and the RNFL thickness change maps were automatically determined by the OCT instrument, with the data then exported to a computer for the purpose of analyzing the progression pattern of the RNFL defects. The RNFL thickness deviation map, which was composed of 50 × 50 pixels, was used to visualize the RNFL defects. When RNFL measurements were below 95% of the percentile range for a particular pixel, yellow was used to code the pixel, while red was used if it was below 99%.

RNFL measurements were performed in each of the subjects at 1 month and 1 day prior to the operation and at 1 and 3 months after the operation. The RNFL thickness change map is one of the components of the GPA software (Carl Zeiss Meditec). This software provides event-based analysis of the RNFL progression based on the serial RNFL thickness maps. Furthermore, as the baseline and follow-up OCT images are automatically aligned and registered, this guarantees that changes at the same pixel locations can be measured. However, in order to generate a GPA report, a minimum of four patient visits is required. Once the images have been obtained and analyzed, the GPA program overlays the images and then compares the serial RNFL thickness versus that obtained during the duration of the follow-up. The event analysis of the GPA was used to assess the RNFL thickness progression. Data obtained from the first two exams were averaged and used as the baseline values. Once the series of RNFL thickness measurements were completed, the baseline RNFL thickness values were compared to the final measurement values via the use of the GPA software. In the current study, the RNFL thickness was defined as having progressed when the RNFL Thickness Map Progression indicated a “likely loss” or “possible loss.”

### 2.3. Visual Field Examination

Each subject underwent standard visual field testing at 1 month and 1 day prior to the operation and at 1 and 3 months after the operation. The visual field testing was performed using static automated white-on-white threshold perimetry (Humphrey Field Analyzer II; Carl Zeiss Meditec, Dublin, CA) with the 30-2 SITA (Swedish Interactive Threshold Algorithm) standard test. Visual fields were only defined as being reliable if the fixation losses and the false-positive and false-negative rates were less than 20%. Only reliable test data were used in our current analyses. When a cluster of three or more points in a pattern deviation plot within a single hemifield with a *P* value < 5% was observed, it was defined as an abnormal visual field. In accordance with the Anderson and Patella criteria, one of these points had to have a *P* value < 1% in order to have the data acceptable [13].

The visual field data determined during the series of follow-up examinations were compared to the patients' baseline visual field data using the GPA software. Data from the first two exams were averaged and used for the baseline values, with the progression evaluation performed relative to the baseline. To evaluate the progression, a database of stable glaucoma patients who were tested over a very short period of time was compared to the observed modifications of the threshold. Fluctuations related to the eccentricity and advancing disease were taken into account for all of the evaluations. To determine if progression had occurred, consecutive visual field tests performed at the same locations (≥3) were examined. In line with the results of a prior study [14], when 2 locations exhibited progression, the GPA printouts defined this as “possible progression,” while “likely progression” was defined when there were 3 locations.

### 2.4. General Anesthesia Procedures

A standardized anesthesia protocol for the drugs was employed during the surgical procedures, with all patients anesthetized with 2–4 *μ*g/ml propofol or 3–5% desflurane. All patients additionally received a continuous infusion of 0.1–1.0 *μ*g/kg/min remifentanil in order to maintain the blood pressure, heart rate, and bispectral index. For pain relief, patients were given remifentanil and fentanyl, while rocuronium was used for muscle relaxation. Mechanical ventilation of the lung was used in order to maintain the end-tidal carbon dioxide (ETCO_2_) concentration at 30–40 mmHg.

A Tono-Pen XL handheld tonometer (Medtronic, Jacksonville, FL) was used to perform the IOP measurements in both eyes of each patient on the day of the operation. Prior to each measurement, the tonometer was calibrated in accordance with the guidelines of the manufacturer. If the variability between sequential measurements exceeded 5%, the measurements were repeated. Measurements of the IOP were performed at 5 min after intubation of the patients who were under systemic anesthesia (T1) and in a supine position, at 6 discrete time points (5, 30, 60, 120, 180, and 240 min; T2-7) after the head was lowered 30 degrees, and at 5 min after returning the patients to a horizontal supine position (T8). The same examiner performed all of the IOP measurements in each subject.

### 2.5. Statistical Analysis

Dunnett's multiple comparison test was used for all of the data analyses. All statistical analyses were performed using SPSS version 19.0 (IBM, New York, NY). A *P* value less than 0.05 was considered to be statistically significant. Data are presented as the mean ± standard deviation.

## 3. Results

Open angles (grade 3 and 4 according to the Shaffer grading system) were observed in all of the patients. After the ophthalmologic examinations, a total of 4 patients with glaucomatous optic disc cupping were excluded. As a result, a total of 40 eyes of 20 subjects, a mean age of 66.9 ± 4.4 years (range, 59 to 72 years), were included in the study. The mean IOP was 15.1 ± 2.0 mmHg at the first visit. The mean visual acuity was −0.09 ± 0.13 log MAR.

**Figure 1:**
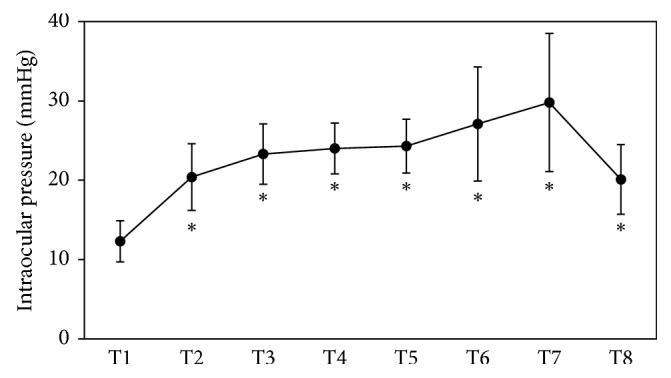
Intraocular pressure (IOP) at each time point. IOP was increased during RALP. ^*∗*^*P* < 0.001 compared with T1.

Mean operation time was 274.4 ± 52.2 min (range: 196–376 min). Mean blood loss was 173.3 ± 158.9 ml (range: 0–452 ml). Mean blood pressure was 109.7 ± 12.0/64.5 ± 9.3 mmHg at T3, 110.0 ± 11.8/66.1 ± 10.5 mmHg at T4, 111.1 ± 12.0/67.9 ± 7.3 mmHg at T5, 100.3 ± 15.5/59.7 ± 10.1 mmHg at T6, and 101.4 ± 11.4/58.2 ± 11.5 mmHg at T7. Mean IOP was 12.3 ± 2.6 mmHg (range: 8–21 mmHg; *n*=40) at T1, 20.4 ± 4.2 mmHg (range: 13–30 mmHg; *n*=40) at T2, 23.3 ± 3.8 mmHg (range: 16–33 mmHg; *n*=40) at T3, 24.0 ± 3.2 mmHg (range: 18–30 mmHg; *n*=40) at T4, 24.3 ± 3.4 mmHg (range: 19–33 mmHg; *n*=38) at T5, 27.1 ± 7.2 mmHg (range: 20–53 mmHg; *n*=24) at T6, 29.8 ± 8.7 mmHg (range: 23–49 mmHg; *n*=8) at T7, and 20.1 ± 4.4 mmHg (range: 12–31 mmHg; *n*=40) at T8 (Figure 1). IOP was significantly increased at all points (T2-8) as compared to T1 (*P* < 0.001).

No abnormal findings were observed before and after RALP for the peripapillary RNFL thickness. After the RALP, there was no progression observed for the RNFL thickness, with a mean RNFL thickness of 90.5 ± 10.4 *μ*m at 1 month before surgery, 91.0 ± 10.0 *μ*m at 1 day before surgery, 91.3 ± 10.2 *μ*m at 1 month after surgery, and 90.9 ± 10.2 *μ*m at 3 months after surgery, respectively (Table 1).

No abnormal findings were observed for the visual fields before and after RALP, and there was no visual field progression after the procedure. Mean deviation (MD) was −0.89 ± 1.08 dB at 1 month before surgery, −0.85 ± 0.74 dB at 1 day before surgery, −0.54 ± 1.09 dB at 1 month after surgery, and −0.40 ± 0.72 dB at 3 months after surgery, respectively (Table 1).

## 4. Discussion

Despite reports of increases in the IOP during the surgery in normal subjects, our current study demonstrated that there were no changes in the values for the visual field and RNFL thickness observed at 1 month and 1 day prior to the RALP procedure and at 1 and 3 months after the RALP procedure. In addition, we used the GPA software to evaluate the effect of the steep Trendelenburg positioning on the visual structure and function. To the best of our knowledge, this is the first study to use statistical analysis to examine the effect of the steep Trendelenburg positioning on the visual structure and function.

Of particular interest is the significant range of IOP elevation that was noted between the patients for each of the measurement time points. At 180 minutes into the procedure, readings of 20 to 53 mmHg were observed, with 9 of 40 eyes found to be above 30 mmHg during the surgery. None of the patients in this study experienced any ocular complications related to the observed increase in the IOP. However, it should be noted that since this study examined patients with healthy eyes, drastic IOP elevations in individuals with glaucomatous eye would most likely be more severe and could potentially impact the vision.

Taketani et al. previously examined patients without any abnormal findings in their optic nerve head and retina at 1 week after surgery and reported that local visual field defects were detected in 7 of 50 eyes in the lower hemifield, with the visual field eventually recovering to normal within 3 months after the surgery [7]. In the current study, however, we observed a normal visual field in every subject at every measurement time point. Even so, since we did not examine the visual field until 1 month after surgery, the possibility exists that we could have missed some subjects who had an abnormal visual field. Another possible explanation for this discrepancy is that visual field defect might be short-term variability in the study of Taketani et al.

Weber et al. previously reported on the development of posterior ischemic optic neuropathy in a 62-year-old patient who lost 1200 ml of blood during a 6 h 35 min RALP procedure [15]. In the current study, the blood loss in our patients was relatively small (mean of 173 ml and maximum of 452 ml), and there was a relatively short operation time (mean of 274 min and maximum of 376 min). Thus, it could be possible that the visual impairment observed in these types of cases is due to a massive blood loss or longer operation time.

As each patient can have a different blood loss and operation time, it is important to evaluate RNFL thicknesses and the visual field progression. However, in order to determine the progression of the RNFL thickness and visual field in each patient, it is necessary to measure the event analysis at least 4 times or if using trend analysis, then 5 times. To the best of our knowledge, this is the first report that has evaluated the RNFL thicknesses and visual field progression using not only the difference of the mean average before and after the operation but also the difference before and after the operation in each patient.

Glaucoma is a neurodegenerative disease of the optic nerve. Although characterized by accelerated retinal ganglion cell (RGC) death, subsequent axonal loss and optic nerve damage, and eventual visual field loss, patients may present with various stages ranging from undetectable to asymptomatic to symptomatic disease [16]. Kerrigan-Baumrind et al. estimated that, in order for standard automated perimetry (SAP) to determine a statistically significant abnormality, a patient would need to have lost at least 23–35% of their RGCs [17]. Functional changes may be detected in many patients prior to any structural changes. However, a structural abnormality of the optic nerve head (ONH) and RNFL is observed in many cases and can be used for an earlier detection of the manifestation of glaucoma [18]. As objective quantification of the structural characteristics of the ONH and RNFL is now possible through the use of OCT, it is feasible to detect glaucoma at an early stage [19]. Thus, in order to help better understand the mechanism of this neurodegenerative disease, it is important that further OCT and SAP evaluations of the effect of the steep Trendelenburg positioning on the retinal structure and function be undertaken.

There were a few limitations for our current study. First, we found no deleterious effect on the retinal function and structure at 1 and 3 months after surgery in the eyes of our healthy subjects. However, given the level of IOP elevation and the length of follow-up in the current study, a larger number of subjects with longer follow-ups are required in order to determine the potential risk to ocular health from glaucomatous changes. Another limitation is that glaucoma or ocular hypertension patients were excluded from the analysis in the current study. Previous studies have reported that eyes with glaucoma or ocular hypertension do exhibit greater IOP fluctuations in conjunction with postural changes [20–22]. At the present time, we are yet to discover what effect the steep Trendelenburg positioning has on the retinal structure and function in glaucoma or ocular hypertension patients while undergoing the RALP procedure. Another potential limitation of the current study is that the sample size was small.

In conclusion, we demonstrated that there was a significantly increased IOP during the RALP procedure. Despite this IOP increase, there were no significant postoperative changes in the retinal structure and function and there were no complications observed in patients without preexisting ocular diseases.

## Figures and Tables

**Table 1:** Retinal nerve fiber layer thickness and visual field at each measure points.

	Average RNFLT (*μ*m)	*P* value	Average MD (dB)	*P* value
1 M before	90.5 ± 10.4		−0.89 ± 1.08	
1 D before	91.0 ± 10.0		−0.85 ± 0.74	
1 M after	91.3 ± 10.2	0.98	−0.54 ± 1.09	0.21
3 M after	91.3 ± 10.2	0.96	−0.40 ± 0.72	0.06

M: month; D: day; RNFLT: retinal nerve fiber layer thickness; MD: mean deviation; *P* value is compared with 1 D before.

## Data Availability

The data (Tables and Figure) used to support the findings of this study are included within the article.

## References

[1] Ficarra V., Novara G., Fracalanza S. (2009). A prospective, nonrandomized trial comparing robot-assisted laparoscopic and retropublic radical prostatectomy in one European institution. *BJU International*.

[2] Tewari A., Sooriakumaran P., Bloch D. A., Seshadri-Kreaden U., Hebert A. E., Wiklund P. (2012). Positive surgical margin and perioperative complication rates of primary surgical treatments for prostate cancer; a systematic review and meta-analysis comparing retropublic, laparoscopic, and robotic prostatectomy. *European Urology*.

[3] Epstein A. J., Groeneveld P. W., Harhay M. O., Yang F., Polsky D. (2013). Impact of minimally invasive surgery on medical spending and employee absenteeism. *JAMA Surgery*.

[4] Awad H., Santilli S., Ohr M. (2009). The effects of steep Trendelenburg positioning on intraocular pressure during robotic radical prostatectomy. *Anesthesia & Analgesia*.

[5] Hoshikawa Y., Tsutsumi N., Ohkoshi K. (2014). The effect of steep Trendelenburg positioning on intraocular pressure and visual function during robotic-assisted radical prostatectomy. *British Journal of Ophthalmology*.

[6] Raz O., Boesel T. W., Arianayagam M. (2015). The effect of the modified Z Trendelenburg position on intraocular pressure during robotic assisted laparoscopic radical prostatectomy: a randomized, controlled study. *Journal of Urology*.

[7] Taketani Y., Mayama C., Suzuki N. (2015). Transient but significant visual field defects after robot-assisted laparoscopic radical prostatectomy in deep Trendelenburg position. *PLoS One*.

[8] Mondzelewski T. J., Schmitz J. W., Christman M. S. (2015). Intraocular pressure during robotic-assisted laparoscopic procedures utilizing steep Trendelenburg positioning. *Journal of Glaucoma*.

[9] Bushley D. M., Parmley V. C., Paglen P. (2000). Visual field defect associated with laser in situ keratomileusis. *American Journal of Ophthalmology*.

[10] Weiss H. S., Rubinfeld R. S., Anderschat J. F. (2001). Case reports and small case series: LASIK-associated visual field loss in a glaucoma suspect. *Archives of Ophthalmology*.

[11] Flammer J., Drance S. M., Zulauf M. (1984). Differential light threshold. Short- and long-term fluctuation in patients with glaucoma, normal controls, and patients with suspected glaucoma. *Archives of Ophthalmology*.

[12] Artes P. H., O’Leary N., Nicolela M. T., Chauhan B. C., Crabb D. P. (2014). Visual field progression in glaucoma: what is the specificity of the guided progression analysis?. *Ophthalmology*.

[13] Anderson D. R., Patella V. M. (1999). *Automated Static Perimetry*.

[14] Weinreb R. N., Khaw P. T. (2004). Primary open angle glaucoma. *The Lancet*.

[15] Weber E. D., Colyer M. H., Lesser R. L., Subramanian P. S. (2007). Posterior ischemic optic neuropathy after minimally invasive prostatectomy. *Journal of Neuro-Ophthalmology*.

[16] Heijl A., Leske M. C., Bengtsson B., Bengtsson B., Hussein M., Early Manifest Glaucoma Trial Group (2003). Measuring visual field progression in the Early Manifest Glaucoma Trial. *Acta Ophthalmologica Scandinavica*.

[17] Kerrigan-Baumrind L. A., Quigley H. A., Pease M. E., Kerrigan D. F., Mitchell R. S. (2000). Number of ganglion cells in glaucoma eyes compared with threshold visual field tests in the same persons. *Investigative Ophthalmology and Visual Science*.

[18] Kass M. A., Heuer D. K., Higginbotham E. J. (2002). The ocular hypertension treatment study: a randomized trial determines that topical ocular hypotensive medication delays or prevents the onset of primary open-angle glaucoma. *Archives of Ophthalmology*.

[19] Jaffe G. J., Caprioli J. (2004). Optical coherence tomography to detect and manage retinal disease and glaucoma. *American Journal of Ophthalmology*.

[20] Tsukahara S., Sasaki T. (1984). Postural change of IOP in normal persons and in patients with primary-open angle glaucoma and low-tension glaucoma. *British Journal of Ophthalmology*.

[21] Hirooka K., Shiraga F. (2003). Relationship between postural change of the intraocular pressure and visual field loss in primary open-angle glaucoma. *Journal of Glaucoma*.

[22] Kiuchi T., Motoyama Y., Oshika T. (2006). Relationship of progression of visual field damage to postural changes in intraocular pressure in patients with normal-tension glaucoma. *Ophthalmology*.

